# Evaluating the use of novel atherogenicity indices and insulin resistance surrogate markers in predicting the risk of coronary artery disease: a case‒control investigation with comparison to traditional biomarkers

**DOI:** 10.1186/s12944-022-01732-9

**Published:** 2022-11-26

**Authors:** Marjan Mahdavi-Roshan, Mohammad Mozafarihashjin, Nargeskhatoon Shoaibinobarian, Zeinab Ghorbani, Arsalan Salari, Amir Savarrakhsh, Azita Hekmatdoost

**Affiliations:** 1 Department of Clinical Nutrition, School of Medicine, Guilan University of Medical Sciences, Rasht, Iran; 2 Lunenfeld-Tanenbaum Research Institute, Sinai Health System, Ontario, Toronto, Canada; 3 Department of Nutrition, School of Medical Sciences and Technologies, Islamic Azad University, Science and Research Branch, Tehran, Iran; 4 Cardiovascular Diseases Research Center, Department of Cardiology, Heshmat Hospital, School of Medicine, Guilan University of Medical Sciences, Rasht, Iran; 5 Department of Nutrition Research, National Nutrition and Food Technology Research Institute, Faculty of Nutrition and Food Technology, Shahid Beheshti University of Medical Sciences, Tehran, Iran

**Keywords:** Atherosclerosis, Coronary heart disease, Hyperlipidemia, Hyperglycemia, Classic risk factors

## Abstract

**Background:**

Due to the contribution of coronary artery disease (CAD) to serious cardiovascular events, determining biomarkers that could robustly predict its risk would be of utmost importance. Thus, this research was designed to assess the value of traditional cardio-metabolic indices, and more novel atherogenicity indices and insulin resistance surrogate markers in the identification of individuals at risk of CAD.

**Methods:**

A case‒control survey was conducted, in which 3085 individuals were enrolled. Their clinical and biochemical data were gathered at baseline. The investigated indices included the atherogenic index of plasma (AIP), triglyceride-glucose (TyG) index, TyG-body mass index (TyG-BMI), lipoprotein combine index (LCI), cholesterol index (CHOLINDEX), Castelli’s risk indices-I, II (CRI-I, CRI-II), and metabolic score for insulin resistance (METS − IR). To examine the relationship between these variables and CAD risk, multiple regression analyses adjusted for potential confounders were conducted.

**Results:**

Overall, 774 angiographically confirmed CAD patients (mean age = 54 years) were compared with 3085 controls (mean age = 51 years). Higher triglyceride, total cholesterol and fasting blood sugar levels and lower HDL-C levels were related to an elevated risk of CAD (*P-for-trend* < 0.001), while the direct association between increased serum LDL-C concentrations and a greater risk of CAD only became apparent when excluding those with diabetes, and statin users. Among novel indices, greater values of the majority of these markers, including AIP, CRI-I, and -II, CHOLINDEX, LCI, and TyG-index, in comparison to the lower values, significantly elevated CAD risk (*P-for-trend* < 0.001).

**Conclusion:**

According to the current findings, novel atherogenicity indices and insulin resistance surrogate markers, in particular, AIP, CRI-I and II, CHOLINDEX, LCI, and TyG-index, may be useful in predicting CAD risk.

**Supplementary Information:**

The online version contains supplementary material available at 10.1186/s12944-022-01732-9.

## Background

In 2019, Cardiovascular diseases (CVDs) were attributed to nearly 32% of worldwide fatalities, i.e., approximately 17.9 million deaths [1]. These diseases have effectively established a “pandemic” in the world [2]. As a major CVD, coronary artery disease (CAD) has been established as the primary cause of mortality and morbidity, a public health burden, and a major contributor to the decline of general well-being worldwide [3, 4]. CAD is known to be an inflammatory disorder generated as a result of multifactorial and complex processes such as atherosclerosis and atherosclerotic thrombosis of the coronary arteries [5, 6]. Over the course of the past years, numerous mechanisms have been suggested as the underlying pathogenesis of CAD, including accumulated glucose and lipid levels, reduced levels or disturbed function of vasodilator factors (e.g., prostacyclin and nitric oxide (NO)), elevated homocysteine levels, endothelial dysfunction, hypercoagulation, and the thrombogenic hypothesis [7–10]. The lipid theory ascribed atherosclerosis to progressive lipid aggregation in the arterial vessel due to the formation of acid mucopolysaccharide complexes and the imbalance between lipid removal and deposition [11–13]. Of note, lipid accumulation and oxidation in the arteries, development of fatty streak and progression of the atherosclerotic lesion have been acknowledged as certain events accelerating the formation and progression of atherosclerosis and contributing to its complications [11–15]. There is also extensive literature supporting the role of triglycerides and cholesterol esters as the two most important circulating lipids involved in atherosclerosis [13, 16–18]. Likewise, insulin resistance could also advance atherosclerosis progression by disrupting lipid metabolism and triggering endothelial dysfunction [8, 9, 19]. Following these metabolic disturbances, vascular occlusion and atherosclerotic plaque rupture may ensue and ultimately culminate in serious embolic and thrombotic cardiovascular (CV) events, including limb ischemia, angina, myocardial infarction, or sudden death due to a CV cause [20, 21]. In this regard, coronary catheterization with an angiogram has been accredited as the CAD prognosis gold standard examination. However, the invasive nature of the procedure prohibits its use as a screening tool [22]. Accordingly, exploring various risk factors that predict CAD occurrence can help researchers and physicians recognize patients who are at an elevated risk for its complications and may therefore need more extensive assessment or intensive treatment. Identifying such candidate biomarkers may also advance the development of novel effective treatment strategies [22]. Detecting perturbed levels of biomarkers indicative of the main pathophysiological disturbances associated with atherosclerosis and CAD, including dyslipidemia, dysglycemia and insulin resistance, may be potentially effective in decreasing the risk of developing more serious CV events. Conventionally, the measurement of serum levels of atherogenic cardiometabolic factors, such as total cholesterol, fasting blood sugar (FBS), triglycerides, and low-density lipoprotein-cholesterol (LDL-C), and anti-atherogenic biomarkers, such as high-density lipoprotein-cholesterol (HDL-C), has been applied to both research and clinical settings to predict the progression of atherosclerotic lesions [23]. However, these ‘conventional’ markers may not be capable of portraying the extent of atherosclerosis severity, and therefore CAD treatment targets based on the levels of these biomarkers may be suboptimal [23]. For instance, reaching a target goal for LDL-C was thought to be associated with adequate prevention of CV events. However, emerging evidence indicates that a high risk for CV events may persist even after normalizing LDL-C levels [24].

On the basis of these considerations, several recently published epidemiological studies have suggested novel atherogenicity-related biomarkers, including the atherogenic index of plasma (AIP), Castelli’s risk indices-I, II (CRI-I, CRI-II), cholesterol index (CHOLINDEX), lipoprotein combine index (LCI), and surrogate markers for insulin resistance such as triglyceride glucose index (TyG), metabolic score for insulin resistance (METS − IR), and triglyceride glucose-body mass index (TyG-BMI). Such markers can be employed as more informative predictors of CV risk and atherosclerosis severity [25–31]. Nevertheless, as of now, there is conflicting evidence about the screening, diagnostic, and prognostic roles of these indices in regard to CAD, specifically among patients who suffer from multiple metabolic comorbidities. It is possible that unique arrays of these biomarkers will be useful for different CAD subpopulations [32–35]. Thus, this research was designed to assess the value of traditional cardio-metabolic indices, such as triglycerides, FBS, HDL-C, total cholesterol, LDL-C, and more novel atherogenicity indices and insulin resistance surrogate markers, including AIP, CRI-I, CRI-II, LCI, CHOLINDEX, TyG, METS − IR, and TyG-BMI, in the identification of individuals at risk of CAD.

## Methods

### Study population

Data collection for the present retrospective case‒control survey was performed between June 2017 and September 2019. During this time period, approximately 20,000 subjects visited the cardiology outpatient clinic at Dr. Heshmat Hospital in Rasht, whether it was due to having clinical signs and/or symptoms of heart disease or for routine check-ups. These subjects were examined by expert cardiologists to rule in or out cardiac etiologies that could explain their symptomology. It was initially assumed to randomly recruit approximately three sex (assigned at birth)-matched non-CAD individuals for each CAD case. Therefore, the medical records of 4400 individuals, of whom 3300 subjects were identified as free of CAD and 1100 were referred to the catheterization laboratory and diagnosed as angiographically confirmed CAD cases, were reviewed by our study staff. After assessing the eligibility for inclusion in the current research and the availability of these individuals’ laboratory and anthropometric data, approximately 3085 subjects in the non-CAD group were recruited to the control arm (controls), and 774 angiographically confirmed chronic CAD patients were allocated to the case group (cases). For the case group, chronic CAD diagnosis was made in accordance with “*ESC 2019 guidelines for the diagnosis and management of chronic coronary* syndromes” [36]. The CAD cases experienced clinical signs such as chest pain and shortness of breath, and showed abnormal findings according to the carried-out (exercise stress test, and echocardiography) and angiography. The control group consisted of subjects with no clinical or laboratory signs of coronary artery disturbances or atherosclerotic disorders. The diagnosis of CAD for controls was ruled out based on the patients’ symptoms (not having typical signs of angina pectoris) and according to the findings of non-invasive investigations, including exercise stress tests and/or echocardiography.

**Fig. 1:**
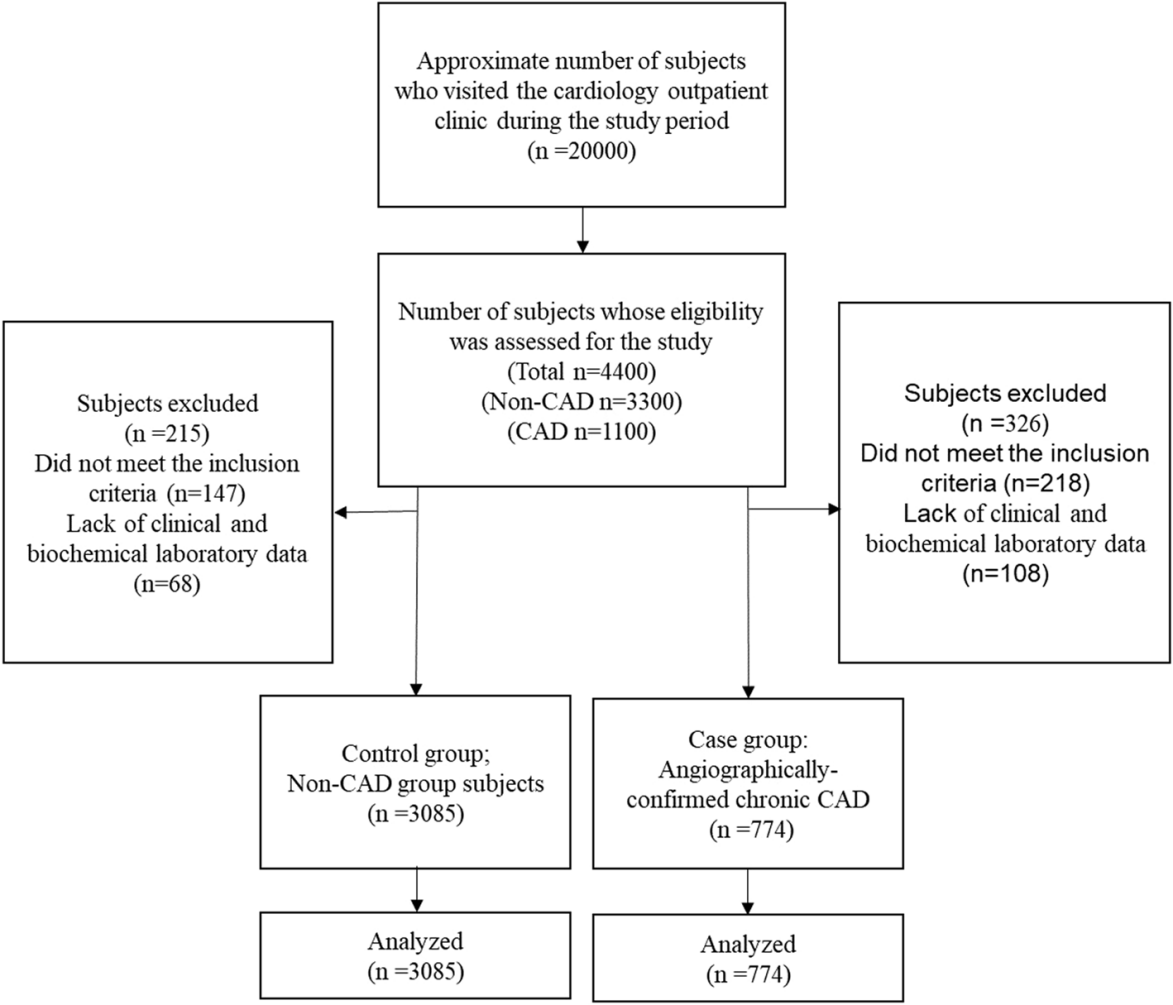
Study participants’ recruitment procedure. CAD, coronary artery disease

To be included in the study, all study participants had to have a BMI between 18.5 and 39 kg/m^2^, and an age between 20 and 75 years old. Conversely, individuals who had a history of unstable angina, cerebrovascular events, myocardial infarction, heart failure, valvular disorders, arrhythmias, cardiomyopathies, malignancies, thyroid dysfunction, acute or chronic liver/kidney diseases, and/or inflammatory/infectious disorders prior to the study or who had previously undergone a coronary angiography (CAG) and/or revascularization procedure were not eligible for the study. Moreover, a lack of clinical and biochemical laboratory data relevant to the participant’s metabolic profile, including classic lipid parameters, glycemic indices or anthropometric measures (e.g., height and weight), was also considered as an exclusion criterion. The study participants’ recruitment procedure is depicted in Fig. 1.

## Clinical data collection

Study staff who were professional healthcare providers reviewed all patient medical records that were relevant to the study. At baseline, demographic and clinical features of participants and their medication consumption history were obtained. Information on anti-hyperlipidemia drugs, particularly statin medications (mainly atorvastatin, simvastatin, and rosuvastatin), was also collected. Considering anthropometric measurements, patients’ weight and height were determined through a Seca 755 medical scale and a standard stadiometer (rounded to the nearest 0·5 kg and 0.1 cm, in order), respectively. BMI was then computed by dividing the weight in kilograms by height in meters squared.

The study procedures were executed on the basis of the guidelines outlined in the 2013 version of the Declaration of Helsinki. The protocol of the current investigation was evaluated and confirmed by the Cardiovascular Diseases Research Center institutional review board, affiliated with Guilan University of Medical Sciences (GUMS) and Ethics Committee of GUMS (registered with research number = 4258, and ethics code = “IR.GUMS.REC.1401.173”).

## Coronary angiography and echocardiography

All the subjects in the case arm of the study were examined by two interventional cardiologists to perform CAG. CAG was performed on the basis of the Judkins technique with 6F catheters by the femoral artery approach. Then, the abovementioned cardiologists, who were blinded to the research protocol, interpreted the angiogram findings, including atherosclerosis severity and level of stenosis. If the narrowing of the vessel was < 50%, stenosis was described as minimal, while a vessel diameter reduction between 50 and 70% was described as moderate stenosis. A diameter decrease of greater than 70% was considered severe (or significant) stenosis. The presence of stenosis in one, two or three major coronary arteries was defined as single-, two- or three-vessel CAD, respectively. Of note, the diagnostic entity of nonobstructive CAD was considered when no culprit lesion was found on the angiogram, and luminal stenosis of epicardial vessels was estimated to be less than 50% [37].

A certified echocardiographer conducted echocardiograms on all CAD patients by means of a standard commercial ultrasound machine and within 72 h of admission to the hospital to estimate left ventricular systolic ejection fraction (LVEF). All echocardiography results were reassessed by two independent cardiologists according to the international Simpson method.

## Laboratory analysis

At the study baseline, venous blood samples (approximately five ml) were obtained following a minimum of 8 h of overnight fasting. The samples were then segregated within 10 min and frozen at − 20 °C until the tests were carried out. Total cholesterol and FBS concentrations were measured by commercial kits (obtained from MAN Co. in Tehran, Iran)) that apply the enzymatic method through cholesterol oxidase and cholesterol esterase, and glucose oxidase enzymes, respectively. HDL-C levels were estimated on the basis of the enzymatic method (MAN Co., Tehran, Iran). An auto Analyzer (Hitachi, Japan) was used to measure these biochemical molecules. The same enzymatic method was used for evaluating triglyceride levels, albeit via glycerol phosphate oxidase and on the Bionic Corporation platform. Ultimately, the Friedewald formula was applied to estimate LDL-C values.

## Explanation of the novel atherogenicity indices and insulin resistance surrogate markers

The formulas that were implemented to calculate the novel atherogenicity and surrogate insulin resistance indices are indicated below: [24, 38–45]

$$\mathrm{Atherogenic}\;\mathrm{index}\;\mathrm{of}\;\mathrm{plasma}\left(\mathrm{AIP}\right)={\mathrm{Log}}_{10}\left(\frac{\mathrm{trglycerides}\left(\frac{\mathrm{mmol}}{\mathrm L}\right)}{\mathrm{HDL}-\mathrm C\left(\frac{\mathrm{mmol}}{\mathrm L}\right)}\right)$$

  

$$\mathrm{Castelli}'\mathrm s\;\mathrm{risk}\;\mathrm{index}-\mathrm I\left(\mathrm{CRI}-\mathrm I\right)=\frac{\mathrm{total}\;\mathrm{cholesterol}\left(\frac{\mathrm{mmol}}{\mathrm L}\right)}{\mathrm{HDL}-\mathrm C\left(\frac{\mathrm{mmol}}{\mathrm L}\right)}$$

  

$$\mathrm{Castelli}'\mathrm s\;\mathrm{risk}\;\mathrm{index}-\mathrm{II}\;\left(\mathrm{CRI}-\mathrm{II}\right)=\frac{\mathrm{LDL}-\mathrm C\left(\frac{\mathrm{mmol}}{\mathrm L}\right)}{\mathrm{HDL}-\mathrm C\left(\frac{\mathrm{mmol}}{\mathrm L}\right)}$$

  

$$\mathrm{Lipoprotien}\;\mathrm{combine}\;\mathrm{index}\;\left(\mathrm{LCI}\right)=\frac{\mathrm{total}\;\mathrm{cholesterol}\left(\frac{\mathrm{mmol}}{\mathrm L}\right)\times\mathrm{triglycerides}\left(\frac{\mathrm{mmol}}{\mathrm L}\right)\times\mathrm{LDL}-\mathrm C\left(\frac{\mathrm{mmol}}{\mathrm L}\right)}{\mathrm{HDL}-\mathrm C\left(\frac{\mathrm{mmol}}{\mathrm L}\right)}$$

$$\mathrm{cholesterol}\;\mathrm{index}\;(\mathrm{CHOLINDEX})=\mathrm{LDL}-\mathrm C\left(\frac{\mathrm{mmol}}{\mathrm L}\right)-\mathrm{HDL}-\mathrm C\left(\frac{\mathrm{mmol}}{\mathrm L}\right)$$

(All patients had triglyceride levels < 400 mg/dL).

$$\mathrm{Metabolic}\;\mathrm{score}\;\mathrm{for}\;\mathrm{insulin}\;\mathrm{resistance}\;(\mathrm{METS}-\mathrm{IR})=\mathrm{Ln}\left(\left(2\times\mathrm{fastingglocuse}\left(\frac{\mathrm{mg}}{\mathrm{dl}}\right)\right)+\mathrm{fastingtriglycerides}\left(\frac{\mathrm{mg}}{\mathrm{dl}}\right)\times\frac{\mathrm{BMI}\left(\frac{\mathrm{kg}}{\mathrm m2}\right)}{\left(\mathrm{Ln}\left(\mathrm{HDL}-\mathrm C\left(\frac{\mathrm{mg}}{\mathrm{dl}}\right)\right.\right)}\right)$$

$$\mathrm{Triglyceride}\;\mathrm{glucose}\;\mathrm{index}\;(\mathrm{TyG})=\mathrm{Ln}\left(\frac{\mathrm{fastingtriglycerides}\left(\frac{\mathrm{mg}}{\mathrm{dL}}\right)\times\mathrm{fastingglucos}\left(\frac{\mathrm{mg}}{\mathrm{dL}}\right)}2\right)$$

$$\mathrm{Triglyceride}\;\mathrm{glocuse}\;-\mathrm{body}\;\mathrm{mass}\;\mathrm{index}\;(\mathrm{TyG}-\mathrm{BMI})=\left(\mathrm{TyGindex}\times\mathrm{BMI}\right)$$

## Statistical analyses

To verify whether the collected data were normally distributed, the Kolmogorov–Smirnov test was used. Continuous variables were described as the mean ± standard deviation (SD). The differences in the mean values of continuous factors between the studied groups were then determined via independent samples T tests. Regarding categorical factors, corresponding data were demonstrated as frequency and percentage, and the Chi-square or Fisher's exact tests were run to compare groups. Comparisons of the traditional markers and novel indices, according to CAG-derived clinical features of CAD patients (i.e., type of CAD and severity of atherosclerosis), were made by applying one-way analysis of variance (ANOVA) followed by Least Significant Difference (LSD) post hoc tests.

Crude and adjusted logistic regression analyses were additionally executed to evaluate the relationship between the targeted variables and CAD risk, and the odds ratios (OR, or adjusted odds ratio, aOR) along with the corresponding 95% confidence interval (95% CI) were estimated for the relationship between independent variables and CAD risk. To test for linear trends across quartiles of lipid parameters and atherogenic indices in relation to CAD risk, the median value of each quartile was considered as a continuous variable.

The reported *P* values are two-tailed. The tests were regarded as significant if the obtained *P* values were less than or equal to 0.05. The Statistical Package for the Social Sciences (SPSS) software (version 21 (Chicago: SPSS Inc. IBM Corp.) was employed for conducting these analyses.

## Sensitivity analysis

To test the robustness and stability of the present findings, two sensitivity analyses were carried out by excluding the subjects who reported consuming statins (first sensitivity analysis) and those with diabetes (second sensitivity analysis) at study baseline to attenuate the potential effects of these likely confounders.

## Results

### Baseline characteristics

Overall, 3085 non-CAD subjects (42.6% males) and 774 CAD patients (40.2% males) were enrolled in the current study. Table 1 demonstrates the baseline demographic, clinical, and anthropometric features of the study participants. Non-CAD individuals (mean (SD) of age = 51 ± 12) were significantly younger than CAD patients (mean (SD) of age = 54 ± 11) (*P* < 0.001). The BMIs of non-CAD and CAD participants were comparable (27.65 ± 4.63 and 27.62 ± 4.49 kg/m2, respectively). CAD patients were more likely to report a family history of CVDs and a personal history of diabetes and hypertension (*P value* < 0.001). In addition, there tended to be more statin users and more smokers among CAD cases than the controls (*P value* < 0.001). Additionally, the patients diagnosed with CAD had significantly elevated concentrations of triglycerides, total cholesterol, and FBS, and reduced concentrations of HDL-C (*P value* < 0.001). However, LDL-C levels did not differ significantly among controls and CAD patients. Additionally, this group showed greater values of novel atherogicity indices and insulin resistance surrogate markers than the non-CAD controls (*P value* < 0.001) (Table 1).

## Echocardiogram and coronary angiogram findings in subjects diagnosed with CAD

Clinical features derived from CAG and echocardiography procedures conducted for CAD study cases are illustrated in Table 2. The majority of CAD patients (~ 64%) were revealed to have mild systolic dysfunction (i.e., LVEF between 41%-55%). The angiographic findings of CAD cases also showed that approximately 42% and 44% of these patients were diagnosed with nonobstructive CAD and suffered from severe stenosis, respectively (Table 2).

Comparisons of traditional cardio-metabolic factors, atherogenicity indices, and surrogate markers of insulin resistance according to CAD patients’ clinical features that were derived from CAG (i.e., type of CAD and severity of atherosclerosis) are shown in Supplementary Tables 1 and 2. Those with three-vessel coronary disease tended to have higher FBS and TyG, and lower BMI and TyG-BMI levels than patients with nonobstructive CAD. In addition, the CAD patients with severe and moderate stenosis showed higher levels of HDL-C, FBS, and TyG, and lower values of BMI, TyG-BMI, CRI-I and METS-IR than those with minimal stenosis (*P value* < 0.05).

## The relationship between traditional cardio-metabolic indices and CAD risk

The odds ratios (95% CIs) for CAD based on the quartiles of the studied variables are demonstrated in Table 3, using crude and adjusted multiple regression models. According to the crude model, patients in quartiles 2, 3 and 4 of triglycerides showed a 39%, 82% and 165% elevated CAD risk compared to 1^st^ quartile patients, respectively (2^nd^ quartile OR = 1.39, 95% CI: 1.07–1.79; 3^rd^ quartile OR = 1.82, 95% CI: 1.42–2.31; 4^th^ quartile OR = 2.65, 95% CI: 2.09–3.35; *P-for-trend* < 0.001). Following adjusting for age, sex, BMI, smoking, family history of heart disease, HTN, type 2 diabetes mellitus, and statin use, an approximately 66%, 90% and 172% elevated risk of CAD was shown for those in the 2^nd^, 3^rd^, and 4^th^ serum triglyceride quartiles as opposed to 1^st^ quartile patients (2^nd^ quartile aOR = 1.66, 95% CI: 1.16–2.38; 3^rd^ quartile aOR = 1.90, 95% CI: 1.33–2.72; 4^th^ quartile aOR = 2.72, 95% CI: 1.90–3.89; *P-for-trend* < 0.001). Each mmol/L increment in serum triglyceride concentration was also associated with an approximately 98% elevated risk of CAD (aOR = 1.98, 95% CI: 1.65–2.37; *P value* < 0.001). Although no significant associations were found based on the crude regression model, when taking into account the abovementioned potential confounders, the patients in the fourth quartile of total cholesterol demonstrated a higher risk of CAD by approximately 51% compared to those in the lowest quartile (aOR = 1.51 95% CI: 1.08–2.12; *P-for-trend* = 0.031). In addition, approximately 30% and 35% elevations in CAD risk were noted for each mmol/L increment in total cholesterol and LDL-C levels, respectively (aOR = 1.30, 95% CI: 1.15–1.45; aOR = 1.35, 95% CI: 1.12–1.53; *P value* < 0.001). Nevertheless, those in the second quartile of LDL-C levels demonstrated a lower risk of CAD by approximately 40% than those in the lowest quartile (2^nd^ quartile aOR = 0.60, 95% CI: 0.42–0.85; *P-for-trend* = 0.009).

On the other hand, an increased serum level of HDL-C was shown to be protective against CAD risk in both bivariable (4^th^ quartile OR = 0.63, 95% CI: 0.50–0.80; *P-for-trend* = 0.001) and multiple regression analyses (4^th^ quartile aOR = 0.41, 95% CI: 0.29–0.59; *P-for-trend* < 0.001) (Table 3). Each mmol/L increment in serum HDL-C concentration was also linked to an approximately 74% reduced risk of CAD (aOR = 0.26, 95% CI: 0.16–0.41; *P value* < 0.001).

Additionally, when comparing higher to lowest FBS quartiles, CAD risk was demonstrated to be augmented (2^nd^ quartile OR = 1.98, 95% CI: 1.34–2.93; 3^rd^ quartile OR = 5.31 95% CI: 3.72–7.58; 4^th^ quartile OR = 26.29, 95% CI: 18.67–37.03; *P-for-trend* < 0.001). This relationship between FBS quartiles and CAD risk persisted in the adjusted regression models (2^nd^ aOR = 2.81, 95% CI: 1.71–4.62; 3^rd^ quartile aOR = 6.34, 95% CI: 4.00–10.04; 4^th^ quartile aOR = 21.56, 95% CI: 13.54–34.33; *P-for-trend* < 0.001). Furthermore, each mmol/L increment in serum FBS levels was accompanied by an approximately 92% elevated risk of CAD (aOR = 1.92, 95% CI: 1.72–2.14; *P value* < 0.001) (Table 3).

## The relationship between novel atherogenicity and insulin resistance surrogate indices, and CAD risk

Significant relationships between various atherogenicity indices including AIP (2^nd^ quartile OR = 1.98, 95% CI: 1.52–2.58; 3^rd^ quartile OR = 3.00, 95% CI: 2.33–3.88; 4^th^ quartile OR = 3.19, 95% CI: 2.47–4.11; *P-for-trend* < 0.001), CRI-I (2^nd^ quartile OR = 1.27, 95% CI: 1.02–1.59; 4^th^ quartile OR = 1.35, 95% CI: 1.08–1.68; *P-for-trend* = 0.042), LCI (2^nd^ quartile OR = 1.62, 95% CI: 1.28–2.06; 3^rd^ quartile OR = 1.65, 95% CI: 1.31–2.10; 4^th^ quartile OR = 1.95, 95% CI: 1.55–2.47; *P-for-trend* < 0.001), METS − IR (2^nd^ quartile OR = 1.67, 95% CI: 1.31–2.14; 3^rd^ quartile OR = 2.23, 95% CI: 1.75–2.83; 4^th^ quartile OR = 2.00, 95% CI: 1.57–2.55; *P-for-trend* < 0.001), TyG index (2^nd^ quartile OR = 1.68, 95% CI: 1.24–2.26; 3^rd^ quartile OR = 2.96, 95% CI: 2.24–3.92; 4^th^ quartile OR = 7.44, 95% CI: 5.70–9.70; *P-for-trend* < 0.001), and TyG-BMI (2^nd^ quartile OR = 1.73, 95% CI: 1.36–2.20; 3^rd^ quartile OR = 1.84, 95% CI: 1.45–2.37; 4^th^ quartile OR = 1.99, 95% CI: 1.53–2.46; *P-for-trend* < 0.001), and CAD risk were found based on crude regression analyses (Table 3). Considering the multiple regression models adjusted for age, sex, BMI, smoking, family history of heart disease, HTN, type 2 diabetes mellitus, and statin use, parallel results were observed for all of the abovementioned novel biomarkers, except for METS-IR and TyG-BMI. Compared to the lowest quartile, elevated levels of AIP significantly multiplied the odds of CAD by approximately 1.9–3.8 times (2^nd^ quartile aOR = 1.92, 95% CI: 1.32–2.79, 3^rd^ quartile aOR = 3.05, 95% CI: 2.10–4.42, 4^th^ quartile aOR = 3.80, 95% CI; 2.62–5.53; *P-for-trend* < 0.001). In addition, each unit increment in the AIP was accompanied by an approximately 8.92-fold elevated risk of CAD (aOR = 8.92, 95% CI: 5.23–15.23; *P value* < 0.001). Also, those in the 2^nd^ and 4^th^ quartiles of the CRI-I, with regard to the lowest values, showed significantly elevated odds of CAD by approximately 73% and 158% (2^nd^ quartile aOR = 1.73, 95% CI: 1.22–2.45; 4^th^ quartile aOR = 2.58, 95% CI: 1.82–3.66; *P-for-trend* < 0.001). Similarly, those in the highest quartile of the CRI-II showed an approximately 68% greater CAD risk than those in the lowest quartile (4^th^ quartile aOR = 1.68, 95% CI: 1.21–2.34; *P-for-trend* < 0.001). Furthermore, each unit increment in the CRI-I and II was associated with an approximately 1.4-fold increased risk of CAD (aOR = 1.37, 95% CI: 1.26–1.49; aOR = 1.43, 95% CI: 1.30–1.57, respectively; *P value* < 0.001). Moreover, the subjects in the highest quartile of CHOLINDEX indicated an approximately 49% elevation in the risk of CAD compared to the lowest quartile (4^th^ quartile aOR = 1.49, 95% CI: 1.09–2.05; *P-for-trend* < 0.001). Additionally, each unit increment in the CHOLINDEX was linked to approximately 1.5 times greater CAD risk (aOR = 1.44, 95% CI: 1.27–1.62; *P value* < 0.001). In addition, higher LCI levels significantly increased the odds of CAD by nearly 2–3 folds, after controlling the regression models for potential confounders (2^nd^ quartile aOR = 2.19, 95% CI: 1.52–3.16, 3^rd^ quartile aOR = 2.63, 95% CI: 1.82–3.79, 4^th^ quartile OR = 3.24, 95% CI: 2.25–4.66; *P-for-trend* < 0.001). Furthermore, each unit increment in the LCI was accompanied by an approximately 2% increased risk of CAD (aOR = 1.02, 95% CI: 1.01–1.03; *P value* < 0.001).

When taking into account the potential confounders, including age, sex, smoking, family history of heart disease, HTN, type 2 diabetes mellitus, and statin use, in the multiple regression model, greater values of METS − IR showed accelerated odds of CAD by approximately 1.5 times in the second and third quartiles as opposed to the first quartile (2^nd^ quartile aOR = 1.46; 95% CI: 1.03–2.06, 3^rd^ quartile aOR = 1.64, 95% CI: 1.18–2.29). Likewise, raised levels of the TyG index significantly worsened the CAD risk by nearly 1.5–5 folds (2^nd^ quartile aOR = 1.64. 95% CI: 1.10–2.45, 3^rd^ quartile aOR = 2.51, 95% CI: 1.71–3.70, 4^th^ quartile aOR = 4.80, 95% CI: 3.29–6.97; *P-for-trend* < 0.001). Furthermore, each unit increment in the TyG index was linked to approximately 4 times greater CAD risk (aOR = 4.12, 95% CI: 3.20–5.30; *P value* < 0.001). Nevertheless, no significant associations were found between TyG-BMI and CAD risk according to the multiple regression analysis (Table 3).

## Sensitivity analysis

The ORs (and 95% CI) for CAD in the quartiles of traditional and novel atherogenicity markers, and insulin resistance surrogate markers remained consistent, when in the sensitivity analysis were conducted excluding the participants who consumed statins at study baseline (Table 4) or those suffering from diabetes (Table 5) were excluded. Interestingly, the direct association between increased serum LDL-C concentrations and CRI-I values and a greater risk of CAD became more apparent in these sensitivity analyses. Furthermore, although those in the second quartile of LDL-C levels demonstrated a lower risk of CAD in the overall analysis, this association was no longer significant when excluding those who used statins at baseline (OR = 0.66, 95% CI: 0.44–1.00).

## Discussion

Collectively, the present results of a case‒control study on 774 angiographically confirmed CAD patients and 3085 non-CAD subjects highlighted that, after controlling for potential confounders, several novel atherogenicity and insulin resistance surrogate indices, including AIP, CRI-I, CRI-II, CHOLINDEX, LCI, and TyG, and to some extent, METS-IR, can significantly predict the risk of CAD. Additionally, the current results demonstrated that traditional dyslipidemia and hyperglycemia serum/plasma biomarkers, such as triglycerides, total cholesterol, HDL-C, and FBS, were able to independently predict the odds of having CAD, taking into consideration the confounding variables’ effects.

In sensitivity analyses, interestingly, the direct association between increased serum LDL-C concentrations and CRI-I values, and greater risk of CAD became more apparent when excluding those with diabetes and statin users. Furthermore, although those in the second quartile of LDL-C levels demonstrated a lower risk of CAD, this association was no longer significant when excluding those who used statins at baseline.

The present research findings on the relationship between novel atherogenicity indices and surrogate markers of insulin resistance, and CAD risk further support the results of previous research efforts [19, 24–31, 40–42, 46–49]. Treating the studied variables as continuous in the multiple regression analysis revealed that higher levels of “AIP (= log_10_(triglycerides/HDL-C))” were linked to an elevated risk of CAD by nearly 9 times. Additionally, subjects with the greatest values of “CRI-I (= total cholestrol/HDL-C)”, CRI-II (= LDL-C/HDL-C), and CHOLINDEX (= LDL-C-HDL-C) demonstrated higher CAD risk by approximately 1.4–1.5 times. Additionally, higher levels of “LCI (= total cholesterol × triglycerides × LDL-C/HDL-C)” significantly increased the CAD risk by approximately 2%. Cai et al. conducted a case–control study in 2017 on patients with CAD (n = 2936) compared to control subjects (n = 2451), aiming to explore the association between the novel and comprehensive atherogenicity indices, and CAD risk among the Chinese Han population, following CAG [24]. Similar to the current study, they revealed that subjects suffering from CAD demonstrated greater levels of atherogenic lipid markers while having reduced HDL-C concentrations. Novel indices, such as the non-HDL-C to HDL-C ratio, non-HDL-C, total cholesterol to HDL-C ratio (i.e., CRI-I), LDL-C to HDL-C ratio (i.e., CRI-II), LCI and AIP, were all demonstrated to be elevated among patients suffering from CAD. Specifically, it was also mentioned that AIP contributed to a higher risk of CAD regardless of other factors [24]. Additional studies have also indicated that AIP could positively correlate with atherosclerosis severity among subjects diagnosed with CAD [19, 47]. In 2020, Qin et al. conducted an observational study in which they identified AIP as a reliable predictor of post-percutaneous coronary intervention (PCI) prognosis, and developed treatment targets among subjects suffering from type 2 diabetes. They also found that greater levels of AIP (especially > 0.318) were accompanied by a higher risk of developing cerebrovascular adverse events [48]. Several other reports have also found a direct and significant link between AIP and carotid intima-media thickness (as an indirect factor showing atherosclerotic lesion development and progression) [50–52]. AIP has additionally been linked to symptomatic carotid artery stenosis [53]. Interestingly, these relationships seem to persist even after accounting for conventional CV risk markers [52, 53]. In accordance with the current study findings and based on the observations of these researchers, it can be speculated that isolated lipid parameters such as LDL-C cannot be considered as comprehensive and reliable predictors of atherosclerotic lesion severity and patient prognosis.

In terms of alternative markers for insulin resistance, the current study revealed that raised levels of the TyG index **(= **Ln [fasting serum triglycerides × fasting blood glucose/2]) significantly worsened the risk of CAD by nearly fourfold. In addition, those in the second and third quartiles of “METS − IR (= Ln ((2 × fasting blood glucose (mg/dL)) + fasting serum triglycerides (mg/dL)) × (BMI (kg/m2))/((Ln (HDL-C (mg/dL))))” showed an accelerated risk of CAD by approximately 1.5 times. However, analyzing the overall association between METS-IR and TyG-BMI (= TyG × BMI) values, and CAD risk did not detect significant findings when treating the variables as continuous in the multiple regression models.

In line with prior reports [54, 55] and the present study findings, a recently published systematic review documented that among asymptomatic individuals, a greater TyG index might be individualistically linked to a greater incidence of atherosclerotic CVDs, including CAD and stroke [26]. Furthermore, similar to more well-known markers for insulin resistance, such as “homeostasis model assessment for insulin resistance (HOMA-IR)”, these novel biomarkers have also been significantly associated with CVD risk factors, such as hypertension, arterial stiffness, coronary stenosis, metabolic syndrome, type 2 diabetes, and all-cause and/or CV mortality [25, 43–45, 56–59].

It has been suggested in the literature that AIP, as a reliable reflector of plasma atherogenicity status, could be applied as an alternative marker for small dense LDL-C (sdLDL-C) and insulin resistance [42, 48, 49]. Of note, sdLDL-C, an LDL-C subcomponent that tends to have greater potential for being oxidized and making foam cells, has been indicated to better represent the progression of atherosclerotic lesions in CAD. However, due to difficulties in the routine assessment of sdLDL-C, particularly in clinical practice, the use of AIP as a reliable surrogate indicator of sdLDL-C has gained considerable attention in recent years [23, 42, 48, 49, 51, 60, 61]. In addition, hypertriglyceridemia, hyperglycemia, and subsequent insulin resistance further modulate the progression of atherosclerosis [8, 9, 19].

The euglycemic-hyperinsulinemic clamp test has been recognized as the “gold-standard” test for measuring insulin resistance [62]. However, routine use of this test is costly and time consuming, especially in the clinical setting. Therefore, several attempts have been made to identify more efficient methods for measuring insulin resistance [23, 44, 56, 62]. In this context, the TyG index has also been mentioned as a reliable and convenient indicator of insulin resistance [23, 44, 56]. TyG was revealed to capture the status of lipotoxicity and glucotoxicity [44, 56]. Furthermore, the use of METS‐IR, as a valid surrogate measure for sensitivity to insulin that incorporates both laboratory and anthropometric measures, has been additionally taken into consideration to recognize subjects who are more prone to developing type 2 diabetes and metabolic syndrome earlier, which are both established CVD risk indicators [44, 63]. The contribution of insulin resistance to pathogenic mechanisms involved in atherosclerotic disorders such as CAD can be biologically plausible; the accumulation of free fatty acids (FFAs), particularly in the hepatic tissue, following a decrease in the sensitivity to insulin (i.e., insulin resistance) and therefore the lack of lipolysis-inhibitory effects of insulin, may result in an elevation in apoB and very low density lipoprotein cholesterol (VLDL-C) formation and reduction of their clearance. Moreover, HDL-C levels will be decreased. These events, together with hypertriglyceridemia, ultimately promote atheroma development [64]. It is thought that lipoproteins with massive triglyceride concentrations and hyperinsulinemia promote the development and advancement of atherosclerotic lesions by promoting the secretion of reactive oxygen species (ROS), increasing inflammation due to monocytes and proinflammatory and adhesion molecules, and reducing NO release by the endothelium. These processes eventually lead to endothelial dysfunction [65–69].

## Comparisons with other studies and what does the current work add to the existing knowledge

Regarding novel indices of atherogenicity and insulin resistance, the available literature has mostly acknowledged the direct link between augmented AIP values, and CVD risk factors (i.e., insulin resistance, hypertension, obesity, and diabetes) and outcomes. For example, AIP has been primarily connected with the progression of metabolic syndrome, CAD, all-cause mortality, and CV events [24, 26, 40–42, 46–50, 52–55, 70]. Moreover, METS‐IR and TyG have been additionally taken into consideration for the early recognition of subjects at high risk for CVD risk factors, particularly type 2 diabetes, in addition to metabolic syndrome [23, 44, 56, 63]. However, less is understood about the link between CAD and METS − IR, TyG and TyG-BMI, as more straightforward, and less expensive methods for estimating the status of insulin resistance.

Therefore, the importance of investigating more comprehensive and efficient markers to detect those at earlier risk of CAD is well recognized. The present results lend support to the current evidence on the high value of novel indices that were demonstrated to be elevated among patients suffering from CAD [24, 26, 40, 41, 48, 50, 52–55, 70]. It seems that such indices, in particular AIP, CRI-I and II, CHOLINDEX, LCI, TyG and METS-IR, may yield more comprehensive and easy to interpret estimates of high-risk individuals for CAD beyond the isolated lipid biomarkers (i.e., triglycerides, LDL-C, HDL-C, or total cholesterol) and FBS. Thus, they can be considered in the initial screening of populations for the identification of patients at risk of CAD, particularly in clinical practice. Furthermore, modeling CAD patient response to therapeutic approaches, including CAG, drug therapies or PCI, according to their baseline levels of novel atherogenic indices and insulin resistance alternative markers would be of utmost importance. With respect to this, the potential usefulness of these indices as a treatment target in clinical trials could additionally be considered as a particular clinical relevance of these findings.

## Study strengths and limitations

### Strengths

There are a few advantages that should be noted; to our knowledge, this is the first investigation in a sample of Iranian subjects in which the risk of CAD is assessed based on the more novel indices for dyslipidemia and hyperglycemia. Furthermore, CAG was performed for all the included CAD patients in the present investigation by two expert interventional cardiologists who were blinded to the research protocol. Thus, misclassification and under-ascertainment of CAD cases were minimized.

## Limitations

When interpreting the present research findings, a number of limitations should be considered. First, the single-center, retrospective, and cross-sectional nature of the study might have biased the results toward confirming a causal association between CAD risk and the candidate indices. Since no participant follow-up data were available, associations between the novel cardiometabolic indices, and incidence of future acute CV events and related morbidity and mortality could not have been explored. Moreover, due to the lack of data on a number of relevant cardiometabolic variables, including dietary intake, physical activity, and waist circumference, the potentially confounding effect of these variables on the study biomarkers and outcome could not have been investigated. Furthermore, since the control subjects were identified using noninvasive methods and were free from cardiac symptoms, we cannot fully ensure that all were free from coronary atherosclerosis, considering the fact that they did not undergo angiography.

## Conclusion

In conclusion, novel atherogenicity indices and insulin resistance surrogate markers, in particular, AIP, CRI-I and II, CHOLINDEX, LCI, and TyG, might be useful in predicting CAD risk. As these indices have the isolated dyslipidemia and/or hyperglycemia biomarkers built in their formulas, they could possibly yield more robust and comprehensive estimates of CAD risk than the traditional lipid and/or glycemia biomarkers by themselves. However, designing long-term prospective cohort surveys is essential to particularly explain the diagnostic value of such novel indices in the early detection of atherosclerotic CVDs and CAD incidence.

**Table 1:** Baseline characteristics of study participants

		**Study groups**				
		**Controls (n = 3,085)**		**CAD patients (n = 774)**		
**Age**, mean, SD		51	12	54	11	< 0.001
**BMI**, mean, SD		27.65	4.63	27.62	4.49	0.874
**Sex**, count, %	**Male**	1,313	42.6%	311	40.2%	0.123
	**Female**	1,772	57.4%	463	59.8%	
**Past medical history**, count, %						
**Hypertension**		50	1.6%	277	35.8%	< 0.001
**Diabetes**		233	7.6%	257	33.2%	< 0.001
**Family history of heart disease**		23	0.7%	274	35.4%	< 0.001
**Statin Users**		333	10.8%	216	27.9%	< 0.001
**Smoking**		151	4.9%	228	29.5%	< 0.001
**Laboratory data** (mmol/L), mean, SD						
** Triglyceride**		1.41	0.59	1.74	0.82	< 0.001
** Total cholesterol**		4.70	0.92	4.82	1.18	0.001
** Low-density lipoprotein-cholesterol (LDL-C)**		2.89	0.80	2.95	1.11	0.065
** High-density lipoprotein-cholesterol (HDL-C)**		1.14	0.27	1.10	.23	< 0.001
** Fasting blood sugar**		4.92	0.85	6.86	2.90	< 0.001
**Novel Markers** mean, SD						
** Atherogenic index of plasma (AIP)**		0.06	0.24	0.17	0.22	< 0.001
** Castelli risk index-I (CRI-I)**		4.33	1.28	4.62	1.64	< 0.001
** Castelli risk index-II (CRI-II)**		2.68	1.02	2.87	1.46	< 0.001
** Cholesterol index (CHOLINDEX)**		1.75	0.86	1.86	1.16	0.012
** Lipoprotein combine index (LCI)**		20.33	16.53	26.76	27.97	< 0.001
** Metabolic score for insulin resistance (METS − IR)**		42.16	8.50	44.32	8.24	< 0.001
** Triglyceride glucose (TyG) index**		8.52	0.49	8.99	0.60	< 0.001
** Triglyceride glucose (TyG)-BMI**		236.20	45.61	248.68	45.43	< 0.001

*BMI* Body mass index, *CAD* Coronary artery disease

**Table 2:** Echocardiography and coronary angiography findings in participants with coronary artery disease

		**Count**	**Column N %**
**LVEF**	**LVEF ≤ 40%**	173	28.4%
	**LVEF between 41%-55%**	389	63.8%
	**LVEF > 55%**	212	27.3%
**Type of CAD**	**Nonobstructive CAD**	322	41.6%
	**One-vessel coronary disease**	129	16.7%
	**Two-vessel coronary disease**	115	14.9%
	**Three-vessel coronary disease**	208	26.9%
**Severity of atherosclerosis**	**Minimal stenosis**	327	42.7%
	**Moderate stenosis**	109	14.1%
	**Severe stenosis**	338	43.7%

*CAD* Coronary artery disease, *LVEF* Left ventricular ejection fraction

**Table 3:** Odds ratio and accompanying 95% confidence interval for having CAD associated with a continuous increment in traditional cardio-metabolic factors, atherogenicity indices, and surrogate markers of insulin resistance and with the participants categorized into quartiles of these variables

	**Quartiles of markers**				*P-for-trend*
	**1**^**st**^	**2**^**nd**^	**3**^**rd**^	**4**^**th**^	
**Serum traditional cardio-metabolic parameters** (mmol/L)					
**Triglyceride**					
**Continuous** ^a^	1.98 (1.65–2.37)				< 0.001
**Cases/controls**	123/819	169/811	212/777	270/678	
**Median** (mmol/L)	0.77	1.14	1.61	2.27	
**Crude model**	1.00	1.39 (1.07–1.79)	1.82 (1.42–2.31)	2.65 (2.09–3.35)	< 0.001
**Adjusted model** ^a^	1.00	1.66 (1.16–2.38)	1.90 (1.33–2.72)	2.72 (1.90–3.89)	< 0.001
**Total cholesterol**					
**Continuous** ^a^	1.30 (1.15–1.45)				< 0.001
**Cases/controls**	208/744	193/820	159/795	214/726	
**Median** (mmol/L)	3.62	4.34	4.97	5.92	
**Crude model**	1.00	0.84 (0.67–1.04)	0.72 (0.56–0.90)	1.05 (0.84–1.30)	0.716
**Adjusted model** ^a^	1.00	1.26 (0.90–1.79)	1.01 (0.70–1.45)	1.51 (1.08–2.12)	0.031
**Low-density lipoprotein-cholesterol (LDL-C)**					
**Continuous** ^a^	1.35 (1.12–1.53)				< 0.001
**Cases/controls**	264/709	141/807	131/847	238/722	
**Median** (mmol/L)	1.92	2.55	3.11	3.93	
**Crude model**	1.00	0.47 (0.37–0.58)	0.42 (0.32–0.52)	0.88 (0.72–1.08)	0.352
**Adjusted model** ^a^	1.00	0.60 (0.42–0.85)	0.76 (0.53–1.01)	1.32 (0.97–1.81)	0.009
**High-density lipoprotein-cholesterol (HDL-C)**					
**Continuous** ^a^	0.26 (0.16–0.41)				< 0.001
**Cases/noncases**	224/885	175/588	249/830	126/782	
**Median** (mmol/L)	0.85	1.03	1.19	1.47	
**Crude model**	1.00	1.17 (0.94–1.47)	1.18 (0.96–1.45)	0.63 (0.50–0.80)	0.001
**Adjusted model** ^a^	1.00	0.83 (0.60–1.16)	0.79 (0.58–1.06)	0.41 (0.29–0.59)	< 0.001
**Fasting blood sugar**					
**Continuous **^a^	1.92 (1.72–2.14)				< 0.001
**Cases/noncases**	40/939	78/923	181/799	475/424	
**Median** (mmol/L)	4.33	4.72	5.16	6.22	
**Crude model**	1.00	1.98 (1.34–2.93)	5.31 (3.72–7.58)	26.29 (18.67–37.03)	< 0.001
**Adjusted model** ^a^	1.00	2.81 (1.71–4.62)	6.34 (4.00–10.04)	21.56 (13.54–34.33)	< 0.001
**Novel indices**					
**Atherogenic index of plasma (AIP)**					
**Continuous** ^a^	8.92 (5.23–15.23)				< 0.001
**Cases/noncases**	98/869	176/786	245/722	255/708	
**Median**	-0.21	0.00	0.18	0.37	
**Crude model**	1.00	1.98 (1.52–2.58)	3.00 (2.33–3.88)	3.19 (2.47–4.11)	< 0.001
**Adjusted model** ^a^	1.00	1.92 (1.32–2.79)	3.05 (2.10–4.42)	3.80 (2.62–5.53)	< 0.001
**Castelli risk index-I (CRI-I)**					
**Continuous** ^a^	1.37 (1.26–1.49)				< 0.001
** Cases/noncases**	172/792	209/756	175/791	218/746	
** Median**	3.00	3.77	4.57	5.97	
** Crude model**	1.00	1.27 (1.02–1.59)	1.02 (0.81–1.29)	1.35 (1.08–1.68)	0.042
**Adjusted model** ^a^	1.00	1.73 (1.22–2.45)	1.45 (1.00–2.09)	2.58 (1.82–3.66)	< 0.001
**Castelli risk index-II (CRI-II)**					
** Continuous** ^a^	1.43 (1.30–1.57)				< 0.001
** Cases/noncases**	224/740	182/787	147/816	221/742	
** Median**	1.59	2.23	2.85	3.97	
** Crude model**	1.00	0.76 (0.61–0.95)	0.60 (0.47–0.75)	0.98 (0.76–1.21)	0.992
** Adjusted model **^a^	1.00	0.98 (0.70–1.38)	0.99 (0.69–1.41)	1.68 (1.21–2.34)	< 0.001
**Cholesterol index (CHOLINDEX)**					
** Continuous **^a^	1.44 (1.27–1.62)				< 0.001
** Cases/noncases**	253/713	150/800	131/847	240/725	
** Median**	0.75	1.40	1.99	2.89	
** Crude model**	1.00	0.52 (0.42–0.66)	0.43 (0.34–0.55)	0.93 (0.76–1.14)	0.693
** Adjusted model** ^a^	1.00	0.64 (0.45–0.91)	0.78 (0.54–1.12)	1.49 (1.09–2.05)	< 0.001
**Lipoprotein combine index (LCI)**					
**Continuous** ^a^	1.02 (1.01–1.03)				< 0.001
**Cases/noncases**	135/830	201/753	205/760	233/732	
**Median**	5.73	12.08	21.19	40.35	
**Crude model**	1.00	1.62 (1.28–2.06)	1.65 (1.31–2.10)	1.95 (1.55–2.47)	< 0.001
**Adjusted model **^a^	1.00	2.19 (1.52–3.16)	2.63 (1.82–3.79)	3.24 (2.25–4.66)	< 0.001
**Metabolic score for insulin resistance (METS − IR)**					
**Continuous** ^b^	1.00 (0.98–1.02)				0.824
**Cases/noncases**	124/841	191/773	239/727	220/744	
**Median**	32.89	39.27	44.82	52.87	
**Crude model**	1.00	1.67 (1.31–2.14)	2.23 (1.75–2.83)	2.00 (1.57–2.55)	< 0.001
**Adjusted model** ^b^	1.00	1.46 (1.03–2.06)	1.64 (1.18–2.29)	1.11 (0.78–1.57)	0.668
**Triglyceride glucose (TyG) index**					
**Continuous** ^b^	4.12 (3.20–5.30)				< 0.001
**Cases/noncases**	77/889	122/841	197/768	378/587	
**Median**	7.97	8.43	8.79	9.22	
**Crude model**	1.00	1.68 (1.24–2.26)	2.96 (2.24–3.92)	7.44 (5.70–9.70)	< 0.001
**Adjusted model** ^b^	1.00	1.64 (1.10–2.45)	2.51 (1.71–3.70)	4.80 (3.29–6.97)	< 0.001
**Triglyceride glucose (TyG)-BMI**					
**Continuous** ^b^	1.00 (0.99–1.02)				0.460
**Cases/noncases**	130/834	205/760	215/750	224/741	
**Median**	185.33	221.36	250.02	293.42	
**Crude model**	1.00	1.73 (1.36–2.20)	1.84 (1.45–2.37)	1.99 (1.53–2.46)	< 0.001
**Adjusted model** ^b^	1.00	1.41 (1.00–1.97)	1.32 (0.94–1.85)	0.86 (0.60–1.22)	0.212

The multiple regression models were adjusted for:

^a^age, sex, BMI, smoking, family history of heart disease, hypertension, type 2 diabetes mellitus, and statin use

^b^age, sex, smoking, family history of heart disease, hypertension, type 2 diabetes mellitus, and statin use

*CAD* Coronary artery disease, *BMI* Body mass index

**Table 4:** Sensitivity analyses of the relationship between CAD risk and traditional cardio-metabolic factors, atherogenicity indices, and surrogate markers of insulin resistance excluding statin users

	**Quartiles of markers**				***P-for-trend***
	**1**^**st**^	**2**^**nd**^	**3**^**rd**^	**4**^**th**^	
**Serum traditional cardio-metabolic parameters** (mmol/L)					
**Triglyceride**					
**Continuous** ^a^	1.97 (1.60–2.43)				< 0.001
**Adjusted model** ^a^	1.00	1.58 (1.04–2.38)	1.79 (1.19–2.69)	2.57 (1.71–3.86)	< 0.001
**Total cholesterol**					
**Continuous** ^a^	1.38 (1.20–1.60)				< 0.001
**Adjusted model** ^a^	1.00	1.25 (0.85–1.86)	1.03 (0.70–1.53)	1.67 (1.15–2.43)	0.011
**Low-density lipoprotein-cholesterol (LDL-C)**					
**Continuous** ^a^	1.55 (1.33–1.81)				< 0.001
**Adjusted model** ^a^	1.00	0.66 (0.44–1.00)	0.86 (0.58–1.28)	1.76 (1.23–2.52)	< 0.001
**High-density lipoprotein-cholesterol (HDL-C)**					
**Continuous** ^a^	0.16 (0.09–0.28)				< 0.001
**Adjusted model** ^a^	1.002003	0.68 (0.46–1.00)	0.66 (0.46–0.94)	0.31 (0.21–0.47)	< 0.001
**Fasting blood sugar**					
**Continuous** ^a^	1.91(1.69–2.16)				< 0.001
**Adjusted model** ^a^	1.00	2.23 (1.26–3.96)	4.72 (2.77–8.04)	19.15 (11.40–32.18)	< 0.001
**Novel indices**					
**Atherogenic index of plasma (AIP)**					
**Continuous** ^a^	12.17 (6.44–23.00)				< 0.001
**Adjusted model** ^a^	1.00	1.94 (1.25–3.00)	3.09 (2.01–4.75)	4.25 (2.75–6.57)	< 0.001
**Castelli risk index-I (CRI-I)**					
**Continuous** ^a^	1.51 (1.37–1.66)				< 0.001
**Adjusted model** ^a^	1.00	1.82 (1.20–2.76)	1.87 (1.22–2.87)	3.63 (2.40–5.50)	< 0.001
**Castelli risk index-II (CRI-II)**					
**Continuous** ^a^	1.64(1.46–1.84)				< 0.001
**Adjusted model** ^a^	1.00	1.07 (0.72–1.59)	1.12 (0.74—1.68)	2.33 (1.59–3.40)	< 0.001
**Cholesterol index (CHOLINDEX)**					
**Continuous** ^a^	1.70 (1.47–1.98)				< 0.001
**Adjusted model** ^a^	1.00	0.64 (0.43–0.96)	1.00 (0.67–1.49)	1.95 (1.36–2.81)	< 0.001
**Lipoprotein combine index (LCI)**					
**Continuous** ^a^	1.03 (1.02–1.03)				< 0.001
**Adjusted model** ^a^	1.00	2.59 (1.68–3.99)	2.57 (1.67–3.97)	4.34 (2.82–6.65)	< 0.001
**Metabolic score for insulin resistance (METS − IR)**					
**Continuous** ^b^	1.00 (0.98–1.02)				0.607
**Adjusted model **^b^	1.00	1.49 (1.00–2.23)	1.67 (1.13–2.46)	1.15 (0.77–1.73)	0.584
**Triglyceride glucose (TyG) index**					
**Continuous** ^b^	4.02 (3.00–5.37)				< 0.001
**Adjusted model **^b^	1.00	1.78 (1.12–2.80)	2.37 (1.52–3.72)	4.48 (2.90–6.92)	< 0.001
**Triglyceride glucose (TyG)-BMI**					
**Continuous** ^b^	0.99 (0.98–1.00)				0.352
**Adjusted model** ^b^	1.00	1.45 (0.99–2.14)	1.17 (0.79–1.73)	0.80 (0.53–1.20)	0.107

The multiple regression models were adjusted for:

^a^age, sex, BMI, smoking, family history of heart disease, hypertension, type 2 diabetes mellitus, and statin use

^b^age, sex, smoking, family history of heart disease, hypertension, type 2 diabetes mellitus, and statin use

*CAD* Coronary artery disease, *BMI* Body mass index

**Table 5:** Sensitivity analyses of the relationship between CAD risk and traditional cardio-metabolic factors, atherogenicity indices, and surrogate markers of insulin resistance excluding those with diabetes

	**Quartiles of markers**				***P-for-trend***
	**1**^**st**^	**2**^**nd**^	**3**^**rd**^	**4**^**th**^	
**Serum traditional cardio-metabolic parameters** (mmol/L)					
**Triglyceride**					
**Continuous** ^a^	2.22 (1.80–2.74)				< 0.001
**Adjusted model** ^a^	1.00	1.46 (0.97–2.20)	1.92 (1.28–2.88)	3.17 (2.13–4.72)	< 0.001
**Total cholesterol**					
**Continuous** ^a^	1.38 (1.21–1.58)				< 0.001
**Adjusted model** ^a^	1.00	1.34 (0.91–1.98)	1.17 (0.77–1.78)	1.80 (1.23–2.62)	0.004
**Low-density lipoprotein-cholesterol (LDL-C)**					
**Continuous** ^a^	1.44 (1.25–1.66)				< 0.001
**Adjusted model** ^a^	1.00	0.57 (0.38–0.85)	0.86 (0.57–1.30)	1.57 (1.10–2.23)	0.001
**High-density lipoprotein-cholesterol (HDL-C)**					
**Continuous** ^a^	0.22 (0.13–0.38)				< 0.001
**Adjusted model** ^a^	1.00	0.82 (0.56–1.19)	0.70 (0.49–0.99)	0.37 (0.25–0.54)	< 0.001
**Fasting blood sugar**					
**Continuous** ^a^	2.72 (2.31–3.22)				< 0.001
**Adjusted model** ^a^	1.00	2.70 (1.55–4.68)	4.47 (2.68–7.44)	21.00 (12.98–33.98)	< 0.001
**Novel indices**					
**Atherogenic index of plasma (AIP)**					
**Continuous** ^a^	11.88 (6.50–21.71)				< 0.001
**Adjusted model** ^a^	1.00	2.06 (1.35–3.15)	2.87 (1.89–4.37)	4.17 (2.74–6.34)	< 0.001
**Castelli risk index-I (CRI-I)**					
**Continuous** ^a^	1.44 (1.32–1.58)				< 0.001
**Adjusted model** ^a^	1.00	1.79 (1.19–2.68)	1.93 (1.27–2.92)	3.27 (2.20–4.87)	< 0.001
**Castelli risk index-II (CRI-II)**					
**Continuous** ^a^	1.51 (1.35–1.68)				< 0.001
**Adjusted model** ^a^	1.00	1.06 (0.72–1.57)	1.31 (0.87–1.96)	2.12 (1.46–3.09)	< 0.001
**Cholesterol index (CHOLINDEX)**					
**Continuous** ^a^	1.54 (1.34–1.77)				< 0.001
**Adjusted model** ^a^	1.00	0.70 (0.47–1.05)	1.01 (0.66–1.53)	1.85 (1.28–2.66)	< 0.001
**Lipoprotein combine index (LCI)**					
**Continuous** ^a^	1.03 (1.02–1.03)				< 0.001
**Adjusted model** ^a^	1.00	2.32 (1.53–3.53)	2.74 (1.80–4.17)	3.87 (2.56–5.85)	< 0.001
**Metabolic score for insulin resistance (METS − IR)**					
**Continuous** ^b^	1.00 (0.98–1.02)				0.707
**Adjusted model **^b^	1.00	1.40 (0.96–2.04)	1.54 (1.07–2.23)	0.94 (0.64–1.39)	0.768
**Triglyceride glucose (TyG) index**					
**Continuous** ^b^	4.44 (3.34–5.92)				< 0.001
**Adjusted model **^b^	1.00	1.85 (1.18–2.89)	2.41 (1.56–3.74)	4.87 (3.22–7.35)	< 0.001
**Triglyceride glucose (TyG)-BMI**					
**Continuous** ^b^	0.99 (0.98–1.00)				0.531
**Adjusted model** ^b^	1.00	1.42 (0.97–2.07)	1.38 (0.95–2.00)	0.88 (0.59–1.29)	0.366

The multiple regression models were adjusted for:

^a^age, sex, BMI, smoking, family history of heart disease, hypertension, type 2 diabetes mellitus, and statin use

^b^age, sex, smoking, family history of heart disease, hypertension, type 2 diabetes mellitus, and statin use

*CAD* Coronary artery disease, *BMI* Body mass index

## Supplementary Information

**Additional file 1:** **Supplementary Table 1**. Comparison of traditional cardio-metabolic factors, atherogenicity indices, and surrogate markers of insulin resistance according to type and severity of atherosclerosis in the studied patients with coronary artery disease.

## Data Availability

The datasets of the current study are available from the corresponding author upon reasonable request.

## Abbreviations

AIP: Atherogenic index of plasma
BMI: Body mass index
CRI-I, CRI-II: Castelli’s risk indices-I,II
CAD: Coronary artery disease
CI: Confidence interval
CAG: Coronary angiography
CHOLINDEX: Cholesterol index
CVDs: Cardiovascular disorders
FBS: Fasting blood sugar
HDL-C: High-density lipoprotein cholesterol
GUMS: Guilan University of Medical Science
HOMA-IR: Homeostatic Model Assessment for Insulin Resistance
sdLDL-C: Small dense low-density lipoprotein cholesterol
LCI: Lipoprotein combine index
LDL-C: Low-density lipoprotein cholesterol
VLDL-C: Very low density lipoprotein cholesterol
METS − IR: Metabolic score for insulin resistance index
BMI: Body mass index
OR: Odds ratio
aOR: Adjusted odds ratio
ROS: Reactive oxygen species
SD: Standard deviation
PCI: Percutaneous coronary intervention
TyG: Triglyceride-glucose index
TyG-BMI: Triglyceride-glucose body mass index
