# Supplementary material for: Evaluating the use of novel atherogenicity indices and insulin resistance surrogate markers in predicting the risk of coronary artery disease: a case‒control investigation with comparison to traditional biomarkers
Source: Lipids Health Dis. 2022 Nov 26;21:126. doi: 10.1186/s12944-022-01732-9 (PMC9701407; doi:10.1186/s12944-022-01732-9)
Supplement: Supplementary file 1 — Additional file 1: Supplementary Table 1. Comparison of traditional cardio-metabolic factors, atherogenicity indices, and surrogate markers of insulin resistance according to type and severity of atherosclerosis in the studied patients with coronary artery disease. [file 12944_2022_1732_MOESM1_ESM.docx]

| **Supplementary Table 1. Comparison of traditional cardio-metabolic factors, atherogenicity indices, and surrogate markers of insulin resistance according to type of coronary artery disease.** | | | | | | | | | |
| --- | --- | --- | --- | --- | --- | --- | --- | --- | --- |
|  | **Type of coronary artery disease** | | | | | | | |  |
|  | **Nonobstructive CAD**  **(n=322)** | | **One-vessel coronary disease**  **(n=129)** | | **Two-vessel coronary disease**  **(n=115)** | | **Three-vessel coronary disease**  **(n=208)** | |  |
|  | **Mean** | **Standard Deviation** | **Mean** | **Standard Deviation** | **Mean** | **Standard Deviation** | **Mean** | **Standard Deviation** | **P value^*^** |
| **Body mass index (BMI)** | 28.32 ^a,b,c^ | 4.83 | 27.31 ^a^ | 4.59 | 27.08 ^b^ | 4.07 | 27.01 ^c^ | 3.95 | 0.003 |
| **Traditional cardio-metabolic parameters (mmol/L)** | | | | | | | | | |
| **Triglyceride** | 1.72 | 0.77 | 1.84 | 0.90 | 1.65 | 0.77 | 1.75 | 0.85 | 0.300 |
| **Total cholesterol** | 4.82 | 1.16 | 4.83 | 1.33 | 4.77 | 1.07 | 4.85 | 1.17 | 0.964 |
| **Low-density lipoprotein-cholesterol (LDL-C)** | 2.93 | 1.11 | 2.88 | 1.14 | 3.00 | 1.08 | 3.01 | 1.11 | 0.678 |
| **High-density lipoprotein-cholesterol (HDL-C)** | 1.11 | 0.23 | 1.12 | 0.24 | 1.08 | 0.21 | 1.07 | 0.24 | 0.087 |
| **Fasting blood sugar** | 6.42 ^a,b^ | 2.35 | 7.07 ^a^ | 3.06 | 6.67 ^c^ | 2.61 | 7.53 ^b,c^ | 3.53 | <0.001 |
| **Novel indices** | | | | | | | | | |
| **Atherogenic index of plasma (AIP)** | 0.16 | 0.21 | 0.18 | 0.23 | 0.15 | 0.21 | 0.18 | 0.22 | 0.507 |
| **Castelli risk index-I (CRI-I)** | 4.53 | 1.52 | 4.54 | 1.77 | 4.62 | 1.49 | 4.80 | 1.81 | 0.305 |
| **Castelli risk index-II (CRI-II)** | 2.79 | 1.36 | 2.74 | 1.44 | 2.96 | 1.44 | 3.04 | 1.61 | 0.157 |
| **Lipoprotein combine index (LCI)** | 25.28 | 23.10 | 29.03 | 34.58 | 25.55 | 22.58 | 28.30 | 32.63 | 0.460 |
| **Cholesterol index (CHOLINDEX)** | 1.82 | 1.15 | 1.76 | 1.18 | 1.92 | 1.14 | 1.94 | 1.16 | 0.420 |
| **Triglyceride glucose (TyG) index** | 8.94 ^a,b^ | 0.56 | 9.07 ^a,c^ | 0.64 | 8.92^c,d^ | 0.53 | 9.07 ^b,d^ | 0.65 | 0.022 |
| **Triglyceride glucose (TyG)-BMI** | 253.48 ^a,b^ | 47.51 | 248.20 | 49.07 | 241.68 ^a^ | 39.26 | 245.42^b^ | 42.38 | 0.050 |
| **Metabolic score for insulin resistance (METS−IR)** | 44.95 | 8.58 | 43.85 | 8.47 | 43.42 | 7.69 | 44.14 | 7.84 | 0.286 |
| ^*^ Obtained from Analysis of variance (ANOVA) test | | | | | | | | | |

| **Supplementary Table 2. Comparison of traditional cardio-metabolic factors, atherogenicity indices, and surrogate markers of insulin resistance according to severity of atherosclerosis.** | | | | | | | |
| --- | --- | --- | --- | --- | --- | --- | --- |
|  | **Severity of atherosclerosis** | | | | | |  |
|  | **Minimal stenosis**  **(n=327)** | | **Moderate stenosis**  **(n=109)** | | **Severe stenosis**  **(n=338)** | |  |
|  | **Mean** | **Standard Deviation** | **Mean** | **Standard Deviation** | **Mean** | **Standard Deviation** | **P value^*^** |
| **Body mass index (BMI)** | 28.61 ^a,b^ | 4.81 | 26.36 ^a^ | 4.00 | 27.06 ^b^ | 4.12 | <0.001 |
| **Traditional cardio-metabolic parameters** (mmol/L) | | | | | | | |
| **Triglyceride** | 1.73 | 0.78 | 1.65 | 0.87 | 1.78 | 0.83 | 0.366 |
| **Total cholesterol** | 4.81 | 1.20 | 4.79 | 1.16 | 4.84 | 1.17 | 0.918 |
| **Low-density lipoprotein-cholesterol (LDL-C)** | 2.95 | 1.16 | 2.90 | 1.05 | 2.97 | 1.08 | 0.834 |
| **High-density lipoprotein-cholesterol (HDL-C)** | 1.07 ^a,b^ | 0.26 | 1.12 ^a^ | 0.22 | 1.11 ^b^ | 0.21 | 0.024 |
| **Fasting blood sugar** | 6.26 ^a,b^ | 2.23 | 6.93 ^a^ | 2.81 | 7.42 ^b^ | 3.36 | <0.001 |
| **Novel indices** | | | | | | | |
| **Atherogenic index of plasma (AIP)** | 0.18 | 0.23 | 0.13 | 0.22 | 0.17 | 0.20 | 0.090 |
| **Castelli risk index-I (CRI-I)** | 4.82 ^a,b^ | 1.94 | 4.43 ^a^ | 1.43 | 4.49 ^b^ | 1.36 | 0.016 |
| **Castelli risk index-II (CRI-II)** | 3.01 | 1.69 | 2.72 | 1.28 | 2.79 | 1.24 | 0.082 |
| **Lipoprotein combine index (LCI)** | 28.02 | 29.20 | 23.00 | 20.25 | 26.75 | 28.86 | 0.268 |
| **Cholesterol index (CHOLINDEX)** | 1.89 | 1.23 | 1.78 | 1.09 | 1.86 | 1.11 | 0.694 |
| **Triglyceride glucose (TyG) index** | 8.92 ^a^ | 0.55 | 8.95 ^b^ | 0.59 | 9.08 ^a,b^ | 0.63 | 0.001 |
| **Triglyceride glucose (TyG)-BMI** | 255.35 ^a^ | 46.89 | 235.99 ^a^ | 40.48 | 246.32 ^a^ | 44.52 | <0.001 |
| **Metabolic score for insulin resistance (METS−IR)** | 45.97 ^a^ | 8.89 | 41.84 ^a^ | 7.10 | 43.53 ^a^ | 7.61 | <0.001 |
| * Obtained from Analysis of variance (ANOVA) test | | | | | | | |

.
